# Protocol of a randomised delayed-start double-blind placebo-controlled multi-centre trial for Levodopa in EArly Parkinson’s disease: the LEAP-study

**DOI:** 10.1186/s12883-015-0491-1

**Published:** 2015-11-19

**Authors:** Constant V. M. Verschuur, S. R. Suwijn, B. Post, M. Dijkgraaf, B. R. Bloem, J. J. van Hilten, T. van Laar, G. Tissingh, G. Deuschl, A. E. Lang, R. J. de Haan, R. M. A. de Bie

**Affiliations:** Department of Neurology, Academic Medical Center, University of Amsterdam, PO BOX 22600, 1100 DD Amsterdam, The Netherlands; Department of Neurology, Donders Institute for Brain, Cognition and Behavior, Radboud University Medical Center, Nijmegen, The Netherlands; Academic Medical Center, Clinical Research Unit, Amsterdam, The Netherlands; Department of Neurology, Leiden University Medical Center, Leiden, The Netherlands; Department of Neurology, University Medical Center Groningen, Groningen, The Netherlands; Department of Neurology, Atrium-Orbis Medical Center Heerlen/Sittard, Heerlen, The Netherlands; Department of Neurology, University Medical Center Schleswig-Holstein, Kiel, Germany; The Edmond J. Safra Program in Parkinson’s Disease and Morton and Gloria Shulman Movement Disorders Center, Toronto Western Hospital, University of Toronto, Toronto, Ontario Canada

**Keywords:** Parkinson’s disease, Levodopa, Randomised delayed-start placebo-controlled trial, Disease modifying

## Abstract

**Background:**

The aim of this study is to investigate if early treatment with levodopa has a beneficial disease modifying effect on Parkinson’s disease (PD) symptoms and functional health, improves the ability to (maintain) work, and reduces the use of (informal) care, caregiver burden, and costs. Additionally, cost-effectiveness and cost-utility of early levodopa treatment will be assessed.

**Methods:**

To differentiate between the direct symptomatic effects and possible disease modifying effects of levodopa, we use a randomised delayed-start double-blind placebo-controlled multi-centre trial design. Patients with early stage PD whose functional health does not yet necessitate initiation of PD-medication will be randomised to either 40 weeks of treatment with levodopa/carbidopa 100/25 mg TID including 2 weeks of dose escalation or to 40 weeks placebo TID. Subsequently, all patients receive levodopa/carbidopa 100/25 mg TID for 40 weeks. There are 8 assessments: at baseline and at 4, 22, 40, 44, 56, 68, and 80 weeks. The primary outcome measure is the difference in the mean total Unified Parkinson’s Disease Rating Scale scores between the early- and delayed-start groups at 80 weeks. Secondary outcome measures are rate of progression, the AMC Linear Disability Score, side effects, perceived quality of life with the Parkinson’s Disease Questionnaire-39, the European Quality of Life-5 Dimensions (EQ-5D), ability to (maintain) work, the use of (informal) care, caregiver burden, and costs. 446 newly diagnosed PD patients without impaired functional health need to be recruited in order to detect a minimal clinical relevant difference of 4 points on the total UPDRS at 80 weeks.

**Discussion:**

The LEAP-study will provide insights into the possible disease modifying effects of early levodopa.

**Trial registration:**

ISRCTN30518857, EudraCT number 2011-000678-72

## Background

Parkinson’s disease (PD) is a neurodegenerative disease characterised by the progression of bradykinesia, tremor, and rigidity, as well as a wide range of non-motor symptoms. The core motor symptoms are caused by the degeneration of dopamine producing neurons [1, 2]. The mainstay of the treatment consists of dopamine replacement either with the dopamine precursor levodopa or with directly acting dopamine receptor agonists (DA).

Although levodopa is inexpensive and very efficacious [3, 4], many neurologists tend to delay initiation and timely adjustments of levodopa. One concern is that levodopa could be toxic, although this has never been supported by the results of clinical studies [5]. Another reason is the concern for side effects such as dyskinesias [1, 6]. Although levodopa-sparing strategies indeed delay the onset of dyskinesias compared to levodopa monotherapy, sooner or later all patients need levodopa [2]. The results of the ELLDOPA study suggest that levodopa may have a beneficial disease modifying effect in addition to the well-known direct symptomatic effect [3]. To date, other studies concerning disease modifying effects of dopaminergic drugs have been inconclusive or negative [7]. More knowledge of possible disease modifying effects of levodopa may improve the use of levodopa in daily clinical practice.

Therefore, we aim to investigate whether the early start of levodopa has a beneficial disease modifying effect on PD symptoms and functional health, subsequently improves patient’s quality of life and the ability to (maintain) work, and reduces the use of (informal) care, caregiver burden and costs. We will also assess cost-effectiveness and cost-utility of early levodopa treatment.

## Methods

### Trial design

**Fig. 1:**
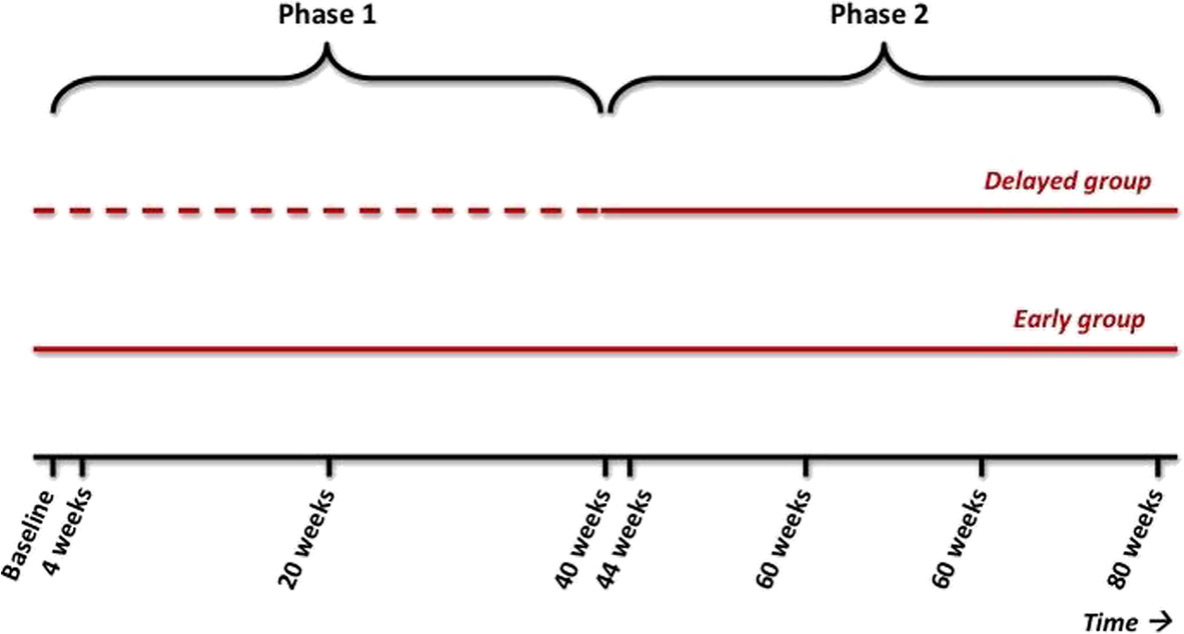
Delayed-start design. Studies with a delayed-start design investigate two agents: active treatment (*solid line*) and controlled treatment (*dashed line*). In phase 1, patients are randomised to either active (levodopa) or controlled (placebo) treatment. In phase 2, both groups receive active treatment

**Fig. 2:**
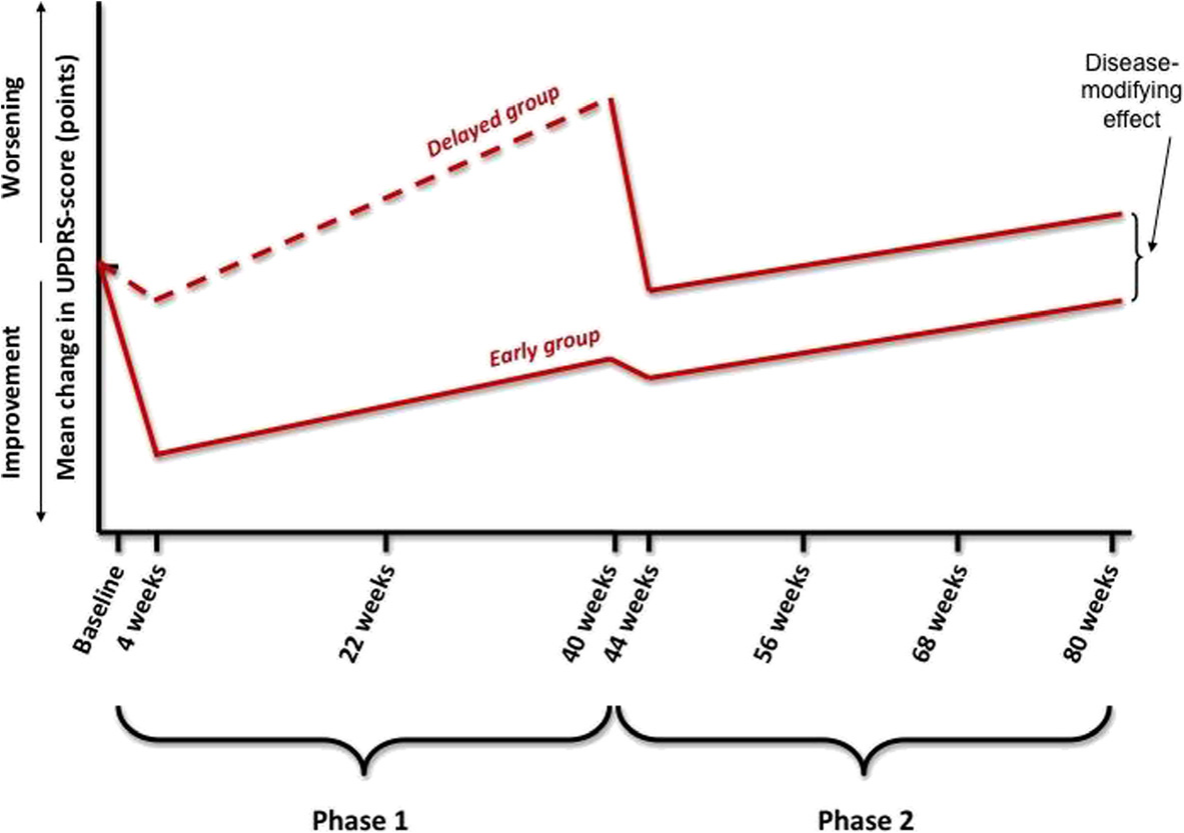
Delayed-start design with beneficial effect of early treatment with levodopa. If a beneficial disease modifying effect of levodopa exists, patients in the early-start group will perform better at the end of the study than patients in the delayed-start group if phase 1 is sufficiently long and if the beneficial disease modifying effect is maintained during phase 2. The small improvement of the delayed-start group at the start of phase 1 and of the early-start group at the start of phase 2 represents the placebo-effect

To differentiate between the direct symptomatic effects and possible disease modifying effects of levodopa, we use a randomised delayed-start double-blind placebo-controlled design (Figs. 1 and 2). Seven academic hospitals and 43 community hospitals in the Netherlands are recruiting patients. Study nurses are trained to perform all study procedures.

### Study participants

The inclusion criteria are (a) idiopathic PD with bradykinesia and at least two of the following signs: resting tremor, rigidity or asymmetry; (b) newly diagnosed PD within the past two years; (c) age 30 years and older; (d) a life expectancy of more than two years; and (e) no limitations in functional health for which the patient needs PD-medication. Exclusion criteria are (a) tremor as most prominent symptom, such as a severe resting tremor that is present almost continuously or a tremor of medium to large amplitude which results in functional disability, such as interfering with feeding; (b) previous treatment with PD-medication; (c) cognitive impairments, defined as a Mini Mental State Examination (MMSE) of 23 points or lower; (d) a score of more than 28 points on the Beck Depression Inventory II (BDI-II); (e) diagnosis of depression by a psychiatrist in the last year; (f) history of psychosis; (g) the presence of signs indicating atypical or secondary parkinsonism; (h) untreated closed-angle glaucoma; (i) alcohol abuse; (j) possible pregnancy; (k) legally incompetent adults, and (l) inability to provide written informed consent.

### Study procedures and randomisation

If a patient is eligible, the neurologist briefly introduces the study and asks the patient permission to send contact information to the coordinating research nurse at the central study hospital (Academic Medical Center, University of Amsterdam). The neurologist provides the patient with written information about the study and informs the research nurse at the central study hospital. Subsequently, the research nurse in the central study hospital registers the patient in the Local Logistics Database. There are five research nurses in five study hospitals based throughout the Netherlands (Academic Medical Center, Atrium-Orbis Medical Center Heerlen/Sittard, Leiden University Medical Center, University Medical Center Groningen, and Radboud University Medical Center). After the patient has been registered, the research nurse based closest to the hospital from where the patient has been put forward is informed. Within three days, the research nurse contacts the patient by phone to answer questions about the study and the nurse makes an appointment to further explain the study if necessary. Patients are required to give a written informed consent to participate.

There are eight assessments, which take place at baseline, four weeks, 22 weeks, 40 weeks, 44 weeks, 56 weeks, 68 weeks, and 80 weeks – respectively Visits 1 to 8. At the end of Visit 1, patients are randomised to 40 weeks treatment with levodopa/carbidopa (early-start group) or to 40 weeks placebo (delayed-start group). The first 40 weeks of the study is called phase 1. Subsequently, all patients receive 40 weeks levodopa/carbidopa – phase 2 (Fig. 1).

Patients in the early-start group take levodopa/carbidopa capsules according to a starting-schedule during weeks 1 and 2, followed by levodopa/carbidopa 100/25 mg tablets TID during weeks 3 to 40 (Table 1). In weeks 41 and 42, they take levodopa/carbidopa 100/25 mg capsules TID. Patients in the delayed-start group take placebo capsules TID during the first 2 weeks, followed by placebo tablets TID during weeks 3 to 40. In weeks 41 and 42, they take levodopa/carbidopa capsules according to a starting-schedule (Table 1). From weeks 43 to 80, all patients receive the levodopa/carbidopa 100/25 mg tablets through their regular pharmacies. In the first 42 weeks of the study, the placebo capsules and tablets are identical in appearance, smell, and taste compared to the levodopa/carbidopa capsules and tablets; they are produced by ACE Pharmaceuticals (Zeewolde, The Netherlands) according to Good Manufacturing Practice guidelines.

**Table 1:** Treatment schedule weeks 1 to 80

		Early group			Delayed group		
		Morning	Noon	Evening	Morning	Noon	Evening
Phase 1	Week 1 (capsules)	50/12.5	Placebo	50/12.5	Placebo	Placebo	Placebo
	Week 2 (capsules)	100/25	50/12.5	50/12.5	Placebo	Placebo	Placebo
	Weeks 3 to 40 (tablets)	100/25	100/25	100/25	Placebo	Placebo	Placebo
Phase 2	Week 41 (capsules)	100/25	100/25	100/25	50/12.5	Placebo	50/12.5
	Week 42 (capsules)	100/25	100/25	100/25	100/25	50/12.5	50/12.5
	Week 43 to 80 (tablets)	100/25	100/25	100/25	100/25	100/25	100/25

Doses are written as levodopa/carbidopa milligram

If the treating neurologist finds that the patient needs additional treatment during the placebo-controlled phase, the patient immediately starts with the capsules initially designated for weeks 41 and 42 and subsequently will use levodopa/carbidopa 100/25 mg TID. This means that - irrespective of initial treatment allocation – the patient is certain to take levodopa/carbidopa from that moment on. Patients that subsequently require additional treatment will receive levodopa/carbidopa 200/50 mg TID. If a patient needs more treatment, the choice and dose of additional medication is left to the discretion of the treating neurologist. If a patient does not follow the study protocol, this is registered; we aim to perform all further procedures and measurements according to the study protocol.

Randomisation is performed by a central website based computer program in 1:1 ratio (Fig. 1) and stratified by type of hospital (university medical centre versus non-university medical centre), age (below 65 years or 65 years and older), and symptom duration (shorter than 0.5 year or 0.5 year and longer), using variable permuted blocks. Study personnel, research nurses, neurologists, and patients are blinded to the treatment allocation at all times. ACE Apothecary (ACE Apotheek, Zeewolde, The Netherlands) allocates the randomised patient to a medication number that corresponds with the treatment group and ACE Apothecary is responsible for packaging, labelling and shipment of the study medication during the first 42 weeks of the study. Randomisation data are kept strictly confidential and are accessible only to authorised persons at ACE Pharmaceuticals and ACE Apothecary until the database is unlocked at the end of the study. All data will be entered in a central website based database before the database is unlocked.

Approval of the medical ethical committee of the Academic Medical Center was obtained. The LEAP-study is conducted according to the principles of the Declaration of Helsinki (version 2008). Study monitoring and data management are performed in accordance with the International Conference on Harmonisation - Good Clinical Practice guidelines. This is an investigator-initiated study. None of the funding organisations has influence on the design or execution of the trial, the analysis of the study data, or the publication of articles concerning the study data.

### Outcome measures

The primary clinical outcome measure is the difference in the mean total Unified Parkinson’s Disease Rating Scale (UPDRS) scores between the early- and delayed-start groups at 80 weeks (Table 2) [8]. Secondary endpoints are (a) progression of symptoms between Visit 2 and Visit 4 (phase 1) and between Visit 5 and Visit 8 (phase 2) measured with the UPDRS; (b) disability measured with the AMC Linear Disability Score (ALDS), defined as the between-group difference at 80 weeks (Table 2) [9]; (c) between-group difference in mean total UPDRS scores at 80 weeks and the progression of UPDRS scores during phases 1 and 2 in patients who followed the study ‘per protocol’; (d) between-group difference in mean total UPDRS scores at 80 weeks and the progression of UPDRS scores during phases 1 and 2 in patients with UPDRS scores in the highest quartile of scores at baseline who followed the study ‘per protocol’; (e) number of patients that need additional medication during phases 1 and 2; (f) cognitive impairment, measured with the MMSE (Table 2) [10]; (g) depression, measured with the BDI-II (Table 2) [11]; (h) perceived quality of life measured with the Parkinson’s Disease Questionnaire-39 (PDQ-39) (Table 2) [12]; (i) quality adjusted life years (QALY), after applying existing scoring algorithms to derive health utilities from observed European Quality of Life-5 Dimensions (EQ-5D) data (Table 2) [13]; (j) working status and absence from paid work measured with a standardised questionnaire; (k) caregiver burden with a standardised questionnaire; (l) resource utilisation outside of the participating hospitals measured with a standardised questionnaire (for (j), (k) and (l) we used an adjusted version of the Short Form - Health and Labour Questionnaire [14, 15] and iMTA Valuation of Informal Care Questionnaire targeted at the study population [16]); (m) costs per unit decrease of the UPDRS and costs per QALY [17, 18]; (n) number of patients withdrawn from the study or lost to follow up; (o) dyskinesias, measured with items 32 to 35 of the UPDRS part IV; (p) levodopa-induced motor response fluctuations throughout the course of the study measured with items 36 to 39 of the UPDRS part IV and three standardised questions concerning wearing-off phenomena (q) (serious) adverse events defined as the frequency, severity, nature, and duration of any adverse event throughout the course of the study.

**Table 2:** Clinical rating scales

Clinical rating scale	Domain	Best score	Worst score
UPDRS	Parkinsonism	0	199
MMSE	Cognition	30	0
BDI-II	Depression	0	63
ALDS	Disability	100	0
EQ-5D	Quality of life	-	-
PDQ-39	Quality of life	0	100

*UPDRS* Unified Parkinson’s Disease Rating Scale, *MMSE* Mini Mental State Examination, *BDI-II* Beck Depression Inventory II, *ALDS* AMC Linear Disability Scale, *EQ-5D* European Quality of Life-5 Dimensions, *PDQ-39* Parkinson’s Disease Questionnaire-39

### Statistics

**Fig. 3:**
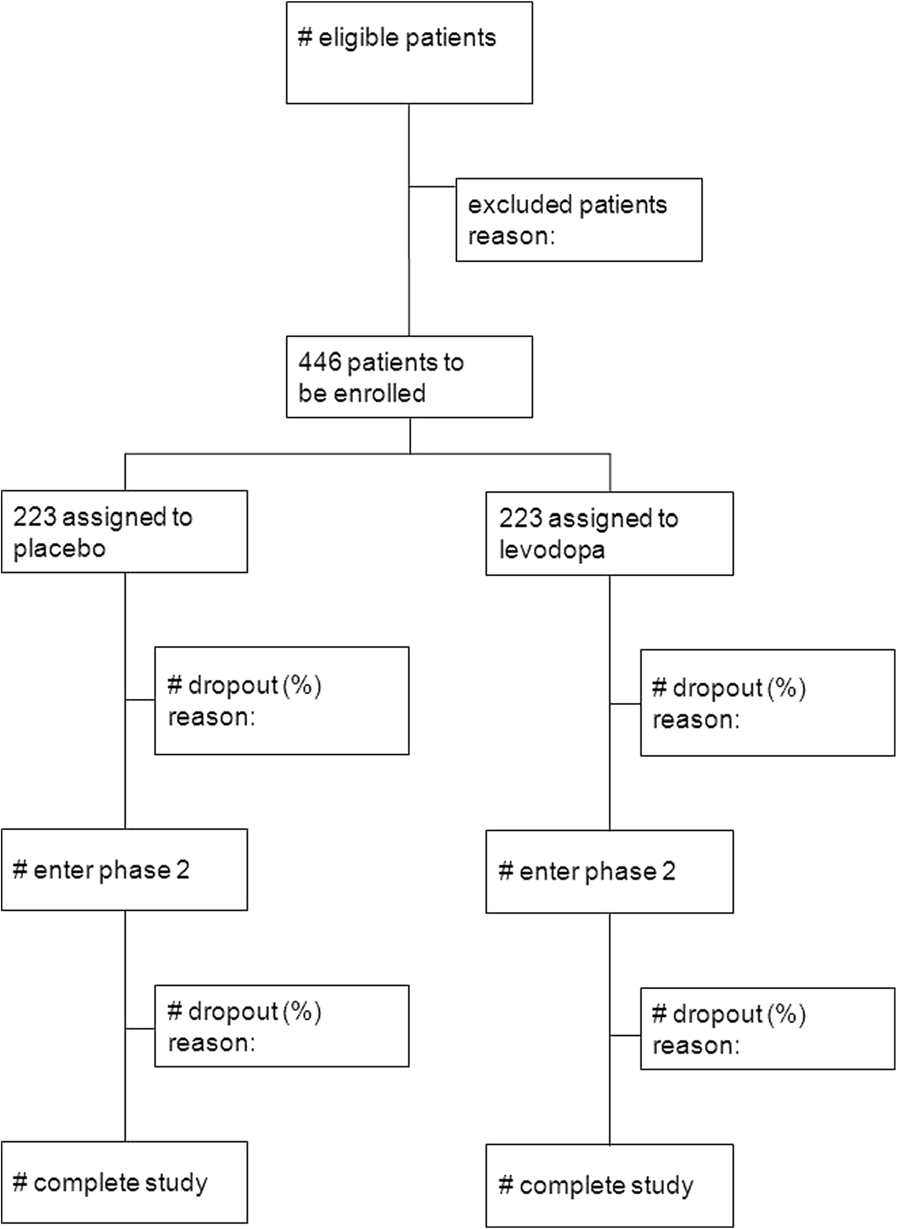
Flow chart LEAP-study. Taking into account a withdrawal rate of 25 %, 446 patients need be enrolled to be able to show a minimal clinically relevant difference of four points on the total UPDRS at the end of the study

Based on the ELLDOPA study, we assume a mean baseline UPDRS score of 28 points with a standard deviation of 13 points and anticipate a mean UPDRS score in the early-start group of 31 points (worsening of 3 points during follow-up) and a mean UPDRS score in the delayed-start group of 35 points (worsening of 7 points) at the final outcome assessment at 80 weeks [3]. Assuming that the common standard deviation is 13, a sample size of 167 in each group will have 80 % power to detect a difference in mean follow-up scores of 4 points, using a two-group *t*-test with a 0.05 two-sided significance level. In the ELLDOPA study, a dropout rate of 22.2 % was reported in the placebo group [3]. After 80 weeks, the ADAGIO-study reported a total dropout rate of 24.4 % in the 1 mg delayed-start group and 19.2 % in the 2 mg delayed-start group. Based on these results, a total withdrawal rate of 25 % was considered realistic [19]: therefore, we plan to include 223 patients per treatment arm, which means 446 patients in total (Fig. 3).

Data will be analyzed according to the intention-to-treat principle. The main analysis of this trial consists of a comparison between the trial treatment groups of the primary outcome after 80 weeks. First, the follow-up difference of mean UPDRS scores between the treatment arms will be crudely analyzed using a two-group *t*-test. Second, the UPDRS follow-up scores will be further investigated using analysis of covariance (ANCOVA), taking into account patients’ UPDRS baseline values. To account for the repeated measures within patients, a random effects model will be used.

With regard to the comparisons of the secondary outcome progression of symptoms during phases 1 and 2 we planned two separate analyses using a random effects model to account for the repeated measures within patients (Fig. 2). For phase 1, we will use the measurements of Visit 2 (4 weeks), Visit 3 (22 weeks) and Visit 4 (40 weeks) to estimate the difference in slopes between the two treatment groups. A more progressive course (steeper upward slope) in the placebo group during phase 1 can reflect disease-modifying effects of levodopa. This will be further explored by examining the slopes of phase 2. Equal steepness of the slope during phase 2 indicates that the (assumed) effect found in phase 1 is a true disease-modifying effect. Therefore, we will use measurements of Visit 5 (44 weeks), Visit 6 (56 weeks), Visit 7 (68 weeks), and Visit 8 (80 weeks) to test equality of progression in phase 2 with a non-inferiority test on the difference between the slopes of the two treatment arms using a non-inferiority margin of 0.055 UPDRS points difference in increase per week (Fig. 2). The slopes of the phases 1 and 2 will be analysed by both an intention-to-treat and a per-protocol approach (see below for further explanation).

As pointed out earlier, the main outcome in this study will be the comparison between treatment arms on UPDRS scores after the total study period of 80 weeks, since this outcome relates to clinical practice. A difference of four points on the total UPDRS is considered to be clinically relevant [20]. The comparisons of slopes in the secondary analyses serve only to differentiate between the different types of treatment effects (direct symptomatic vs disease modifying). Here we pre-specify the possible inference made on these secondary analyses:

1. if a faster progression is found in the placebo group during phase 1 and the non-inferiority assumption assuming equal progression is met in phase 2, this will be indicative of a true disease-modifying effect;
2. if a faster progression is found in the placebo group during phase 1, but non-inferiority of slopes during phase 2 is not demonstrated, then the results are indicative of only direct symptomatic effects.

With regard to the comparisons of the other secondary outcomes (disability, number of patients that need additional medication for PD, cognitive impairment, mood, quality of life, ability to (maintain) work, use of (informal) care, caregiver burden, levodopa-induced response fluctuations, and side effects), we will use the appropriate parametric and nonparametric statistics. Cost-effectiveness and cost-utility analyses will be performed from a societal perspective with the costs per unit decrease on the UPDRS respectively, the costs per QALY as outcomes. In all analyses statistical uncertainties will be expressed in 95 % confidence intervals.

Subgroup analyses by type of hospital (university medical centre versus non-university medical centre), age and disease duration will be performed. Finally, two additional ‘per protocol’ analyses will be carried, focusing on (a) between-group difference in UPDRS scores at 80 week and the progression of UPDRS scores during phases 1 and 2, and (b) between-group difference in UPDRS scores at 80 weeks and the progression of UPDRS scores during phases 1 and 2 in patients in the highest quartile of scores at baseline. Patients will be excluded from the per protocol analysis if they needed additional PD-medication during the study, if they did not use the correct dose and type of medication (e.g., extended release levodopa formulations), or if they missed either Visits 1, 2, 4, 5 or 8.

## Discussion

To differentiate between the direct symptomatic effects and possible disease modifying effects of levodopa, we use a randomised delayed-start double-blind placebo-controlled multi-centre trial design [21]. Some of the choices in the study design warrant discussion.

First, we discuss the stratification for type of hospital, age and disease duration. We stratified by type of hospital (university medical centre versus non-university medical centre) because of possible differing patient characteristics. Stratification by age was chosen because of the different rates of disease progression [22] and because younger patients have a higher risk to develop dyskinesias [23]. Furthermore, younger patients are working more often than older patients, which may bias the cost-effectiveness and cost-utility analyses. Stratification by disease duration was chosen because it is more likely that patients with longer disease duration at the time of inclusion will need the addition of symptomatic medication earlier in the course of the study.

The second relevant issue is the approach we chose for patients that need additional treatment during the placebo-controlled phase of the study. If the treating neurologist decides that a patient needs more treatment, the patient will first start with the capsules initially designated for weeks 41 and 42, after which the patient is certain to be taking levodopa/carbidopa – thereby inducing a placebo effect in patients from the early-start group (which are already on levodopa) and an additional direct symptomatic effect of levodopa in patients from the delayed-start group (which were on placebo) [19]. This procedure prevents unblinding.

Third, the placebo-controlled phase of 40 weeks was chosen based on the results of the ELLDOPA study, which suggested a possible disease modifying beneficial effect of levodopa evident after 40 weeks of treatment [3]. Ideally, the placebo-controlled phase of 40 weeks would even be longer because if a disease modifying effect of levodopa exists, there would then be more time for the slopes of the two treatment groups to diverge further (Fig. 2). However, because of symptom progression, a placebo-controlled phase of more than 40 weeks would result in the retention of progressively fewer patients in the placebo-controlled phase of the study, making the placebo-controlled phase only slightly longer because of the increasing lower numbers in the delayed-start group – thereby diminishing the theoretical advantage of a longer placebo-controlled phase. More importantly, poorer retention in the delayed-start group would compromise the ability to show a difference between the two groups and would potentially select out patients with a milder or more slowly progressive disease course, thereby diminishing a possible existing difference between the two groups. Also, a longer placebo-controlled phase would result in higher costs in this investigator-initiated study. A study design with an additional delayed-stop (at 40 weeks) arm was rejected because this would require patients that typically need antiparkinson medication at this point would experience increased disability and discomfort due to having treatment withheld.

Fourth, we chose a daily levodopa/carbidopa dose of 300/75 mg. In the ELLDOPA study, three levodopa/carbidopa doses (150/37.5 mg, 300/75 mg or 600/150 mg daily) were tested and compared with placebo for 40 weeks, followed by a two-week washout period. The results suggested a beneficial disease modifying effect of levodopa in all treatment groups, thereby making a strong case to choose the 150/37.5 mg daily dose for the current study. However, in the 150/37.5 mg group, there was also a higher dropout rate [3]. We considered a daily treatment of levodopa/carbidopa 600/150 mg too high because of the risk of more side effects [24] and because in daily practice, starting-doses of levodopa are usually lower.

All possible outcomes of the LEAP-study are likely to have clinical implications. If the LEAP-study demonstrates a beneficial disease modifying effect of the early initiation of levodopa (Fig. 2), an argument can be made for starting treatment as soon as the diagnosis of PD is made. Subsequently, functional health and quality of life may improve substantially – thereby prolonging the period of higher social functioning and participation in the workforce. If the study indicates that there is no disease modifying effect of levodopa, it can still be argued that there is also no reason to delay the use of levodopa early in the disease: after all, levodopa has a large symptomatic effect. Important to note here is that motor complications seem to relate more to disease severity and disease duration than treatment duration [25]. The third possible outcome is that the LEAP-study shows a negative disease modifying effect after 40 weeks of follow-up. Of course, this would be a strong argument in favour of levodopa sparing strategies.

The reason to perform two subgroup analyses – the per protocol analysis and the per protocol analysis for the patients with the highest quartile UPDRS-scores at baseline – is to address the possibility that a direct symptomatic effect of levodopa would mask a disease modifying effect in this cohort with relatively mild Parkinson’s disease.

Regardless of these possible outcomes of the LEAP-study, no clear statement can yet be made about the effects of early or delayed starting with levodopa beyond the study period. Some long-term effects of levodopa – such as the development of dyskinesias and response fluctuations – may take years to develop, which may offset any initial beneficial effect evident in the early start group, speaking in terms of total quality-adjusted life years and costs. In this light, the results of the open label PD MED study have to be weighed carefully [2]. This study did not show a difference between the PDQ-39 mobility scores of patients who started treatment with levodopa as compared to patients starting with levodopa-sparing therapy (DA or a Monoamine Oxidase-B inhibitor). A high percentage of patients in the levodopa-sparing arm also received levodopa in different dosages at different times during the follow-up – thereby possibly masking a disease modifying effect of levodopa. Interestingly, the group that started with levodopa used a lower levodopa equivalent dose at the end of the study. This could be due to a beneficial disease modifying effect of early levodopa, but could also be an artifact of the formula used to calculate the levodopa equivalent dose.

## Protocol

The full study-protocol can be accessed through the study website www.leapamc.nl.

## Abbrevations

ALDS: AMC Linear Disability Score
BDI-II: Beck Depression Inventory II
DA: Dopamine receptor agonist
MMSE: Mini Mental State Examination
PD: Parkinson’s disease
PDQ-39: Parkinson’s Disease Questionnaire-39
UPDRS: Unified Parkinon’s Disease Rating Scale
